# Between virus correlations in the outcome of infection across host species: Evidence of virus by host species interactions

**DOI:** 10.1002/evl3.247

**Published:** 2021-07-15

**Authors:** Ryan M. Imrie, Katherine E. Roberts, Ben Longdon

**Affiliations:** ^1^ Centre for Ecology and Conservation, Biosciences, College of Life and Environmental Sciences University of Exeter Penryn TR10 9FE United Kingdom

**Keywords:** Comparative studies, host–parasite interactions, insects, viruses

## Abstract

Virus host shifts are a major source of outbreaks and emerging infectious diseases, and predicting the outcome of novel host and virus interactions remains a key challenge for virus research. The evolutionary relationships between host species can explain variation in transmission rates, virulence, and virus community composition between hosts, but it is unclear if correlations exist between related viruses in infection traits across novel hosts. Here, we measure correlations in viral load of four *Cripavirus* isolates across experimental infections of 45 *Drosophilidae* host species. We find positive correlations between every pair of viruses tested, suggesting that some host clades show broad susceptibility and could act as reservoirs and donors for certain types of viruses. Additionally, we find evidence of virus by host species interactions, highlighting the importance of both host and virus traits in determining the outcome of virus host shifts. Of the four viruses tested here, those that were more closely related tended to be more strongly correlated, providing tentative evidence that virus evolutionary relatedness may be a useful proxy for determining the likelihood of novel virus emergence, which warrants further research.

**Impact Summary**

Many new infectious diseases are caused by viruses jumping into novel host species. Estimating the probability that jumps will occur, what the characteristics of new viruses will be, and how they are likely to evolve after jumping to new host species are major challenges. To solve these challenges, we require a detailed understanding of the interactions between different viruses and hosts, and metrics that can capture some of the variation in these interactions. Previous studies have shown that the evolutionary relationships between host species can be used to predict traits of infections in different hosts, including transmission rates and the damage caused by infection. However, the potential for different viruses to influence the patterns of these host species effects has yet to be determined. Here, we use four viruses of insects in experimental infections across 45 different host species of fruit fly to begin to answer this question. We find similarities in the patterns of replication and persistence between all four viruses, suggesting susceptible groups of related hosts could act as reservoirs and donors for certain types of virus. However, we also find evidence that different viruses interact in different ways with some host species. Of the four viruses tested here, those that were more closely related tended to behave in more similar ways, and so we tentatively suggest that virus evolutionary relatedness may prove to be a useful metric for predicting the traits of novel infections, which should be explored further in future studies.

Virus host shifts, where viruses jump to and establish onward transmission in novel host species, are a major source of outbreaks and emerging infectious diseases (Cleaveland et al. 2001; Taylor et al., 2001; Woolhouse and Gowtage‐Sequeria 2005). Many human infections, including Measles virus, HIV, and recently SARS‐CoV‐2, have shifted into humans from other species and continue to cause significant damage to public health, society, and the global economy. (Frank et al. 2019; Miller et al. 2020; Misin et al. 2020; Shereen et al. 2020). Predicting and preventing virus host shifts have consequently become major goals of virus research (Holmes 2013). Many challenges remain in achieving these goals, including improving our understanding of the host, virus, and ecological factors that influence the outcome of initial cross‐species transmission (Olival et al. 2017; Plowright et al. 2017), and the evolutionary and epidemiological factors that determine which pathogens become established in novel hosts (Geoghegan and Holmes 2017).

Several studies have investigated the ability of host evolutionary relatedness to explain variation in the outcome of infection across host species, where it acts as a proxy for underlying divergence in the immunological and physiological traits that influence host susceptibility. Greater phylogenetic distance between the natural (donor) and recipient hosts is associated with decreased likelihood of cross‐species transmission (Gilbert and Webb 2007; Streicker et al. 2010) and reduced onward transmission within the novel host species (Guth et al. 2019). Additionally, phylogenetic distance between hosts can explain variation in virulence after cross‐species transmission, which increases when viruses jump between more distantly related hosts (Farrell and Davies 2019; Guth et al. 2019; Mollentze et al. 2020). Groups of closely related hosts have also been shown to share similar levels of susceptibility to novel viruses, independent of the distance to the natural host (Longdon et al. 2015; Longdon et al. 2011), and harbor similar virus communities (Davies and Pedersen 2008; Albery et al. 2020; Shaw et al. 2020).

In these studies, variation across host species is measured either with a single virus, or across multiple virus families to detect broad patterns. However, little is known about the potential for individual viruses to interact with host evolutionary effects (Longdon et al. 2011). Within host species, genotype‐by‐genotype interactions between host and virus can be important determinants of the outcome of infection (Hudson et al. 2016), with similar interactions seen in bacterial and *Plasmodium* infections in other systems (Lambrechts et al. 2005; Hall and Ebert 2012). These interactions alter the rank order of host susceptibility and so reduce the strength of correlations in susceptibility to different parasites across hosts. In fungal pathogens of plants (Vienne et al. 2009) and ectoparasites of mammals (Hadfield et al. 2014), comparative analyses have revealed effects of parasite evolutionary relatedness, alongside those of host evolutionary relatedness, and some evidence exists to suggest similar effects may be found in viruses. Closely related viruses tend to infect the same broad host taxa (Kitchen et al. 2011), despite high levels of geographic range overlap between potential hosts (Jenkins et al. 2013), suggesting they share similar constraints on their host ranges. Both co‐speciation and the preferential host switching of viruses can support this, given that viruses are overwhelmingly likely to encounter other host taxa over the timescales required for speciation. That said, shifts between divergent host species are also common across every virus family (Geoghegan et al. 2017) and these exceptions include several human zoonoses of major concern (Guth et al. 2019).

Within virus families, the strength of correlations that exist between viruses in variable infection traits, and how evolutionary relatedness may influence these correlations, has yet to be firmly established. Despite this, it is common, and at times necessary, to infer the characteristics of viruses from better studied relatives. This is frequently the case during the early stages of outbreaks, where primary research on new viruses or variants is not available. When SARS‐CoV‐2 first emerged, its characteristics and epidemiological trajectory were inferred from closely related zoonotic and endemic coronaviruses (Zhu et al. 2020), and from other pandemic respiratory viruses such as influenza A (Petersen et al. 2020). Comparisons to previous outbreaks were used to parameterize disease models in the 2009 H1N1 pandemic (Chao et al. 2010; Hsieh 2010), the 2014 Ebolavirus outbreak (Gomes et al. 2014), and in forecast models of seasonal influenza (Du et al. 2017). Even for viruses that are not newly emerged, many experimental models of infection rely on surrogates when the virus of interest is unavailable, nonpermissive in cell culture or animal models, or requires considerable adaptation to experimental hosts (Cann et al. 2013; Ruiz et al. 2017).

These comparisons assume that the traits of one virus are similar to other, related viruses. However, comparisons between more distantly related viruses, such as bat and canine rabies viruses (Pérez‐Losada et al. 2015) and diverged lineages of influenza viruses (Zanotto et al. 1996; Buchon et al. 2014; Zhang et al. 2019), found stark differences across larger evolutionary scales. Many examples also exist of small genetic changes having large phenotypic effects in viruses, including single SNP changes altering the host range of canine parvoviruses (Allison et al. 2016), the vector specificity of Chikungunya virus (Tsetsarkin et al. 2007), and the infectivity of naturally occurring Ebolaviruses (Wong et al. 2018). Only three amino acid substitutions are required to switch receptor specificity of avian H7N9 influenza from poultry to human cell receptors (de Vries et al. 2017). Virus evolution is often characterized by high mutation rates and frequent reassortment and recombination (Holmes 2009; Pérez‐Losada et al. 2015; Müller et al. 2020). This, alongside an incomplete sampling of extant viruses (Zhang et al. 2019), has left many poorly resolved evolutionary relationships between and within existing virus lineages (Zanotto et al. 1996). Given these complications, it remains an open question whether comparisons between related viruses can produce consistent and accurate inferences of infection traits.

In this study, we have investigated how patterns of host susceptibility (measured here as the ability of a virus to persist and replicate in the host) are correlated between viruses, using experimental infections of four *Cripavirus* isolates (family *Dicistroviridae*) across a panel of 45 host species of *Drosophilidae*. *Drosophila* are a well‐established invertebrate model of innate immunity, responsible for major immunological discoveries including Toll, and possess both Dicer‐mediated antiviral RNAi responses and genotype‐specific immune memory (Buchon et al. 2014; Mussabekova et al. 2017; Mondotte et al. 2020). Three of the viruses tested here are isolates of Drosophila C virus (DCV‐C, DCV‐EB, and DCV‐M), a well‐studied virus isolated from *Drosophila melanogaster* (Johnson and Christian 1999), and represent the most divergent available isolates of this virus species. The fourth virus is the closely related Cricket Paralysis virus (CrPV), which was isolated from Australian field crickets (*Teleogryllus commodus*) and is a widely used model insect pathogen (Reinganum et al. 1970; Cherry and Silverman 2006; Bonning and Miller 2010).

DCV is known to naturally infect at least two *Drosophila* species in the wild—*D. melanogaster* and *D. simulans* (Comendador et al. 1986; Kapun et al. 2010)—whereas CrPV is not known to naturally infect any *Drosophila* species (Christian and Scotti 1998). Despite this, both DCV and CrPV are shown to be capable of infecting a broad range of insect taxa in experimental studies (Scotti et al. 1981). Both cause virulent infections in adult flies (Longdon et al. 2015; Nayak et al. 2018) and share similar mechanisms for co‐opting the host translation machinery (Majzoub et al. 2014). A major‐effect resistance gene called *pastrel* increases resistance to DCV in *D. melanogaster* (Magwire et al. 2012; Cogni et al. 2016; Cao et al. 2017) and has also been shown to provide cross‐resistance to CrPV along with another gene, *Ubc‐E2H* (Martins et al. 2014). Both DCV and CrPV are targeted by the host antiviral RNAi pathway and each encodes a potent suppressor of antiviral RNAi. However, these suppressors have different functions and target different components of the RNAi pathway (van Rij et al. 2006; Nayak et al. 2010). DCV and CrPV also differ in their tissue pathology; DCV has been shown to infect gut tissues, causing intestinal obstruction following septic inoculation in *D. melanogaster*, which was not observed in CrPV infection (Chtarbanova et al. 2014). Although little is known about the differences between DCV isolates, they have been shown to cause similar levels of virulence in *D. melanogaster* (Martinez et al. 2019).

Previous work in this host system has shown that susceptibility to DCV‐C varies across host species, that the host phylogeny explains a large proportion of the variation in both viral load and virulence, and that viral load and virulence are strongly positively correlated (Longdon et al. 2015). The host phylogeny is also an important determinant of the evolution of DCV‐C in novel hosts, with evidence that mutations that adapt the virus to one host may also adapt it to closely related host species. This suggests virus genotype could alter the likelihood of host shifts in *Drosophila* (Longdon et al. 2018). Here, we measure correlations in the ability of four viruses to replicate and persist across host species and provide evidence of both broad similarities in infection outcome and differences consistent with virus by host species interactions.

## Materials and Methods

### FLY STOCKS

Flies were taken from laboratory stocks of 45 different species of *Drosophilidae* (for details, see Table SA). Before experiments began, all included stocks were confirmed to be negative for infection with DCV and CrPV by quantitative reverse transcription PCR (qRT‐PCR, described below). Stocks were maintained in multigeneration *Drosophila* stock bottles (Fisherbrand) at 22°C, in a 12‐hour light‐dark cycle. Each bottle contained 50 ml of one of four varieties of food media (Supporting Information Methods), which were chosen to optimize rearing conditions of parental flies. Changes in the macronutrients available to adult *Drosophila* have been shown to have little effect on the outcome of viral infection (Roberts and Longdon 2021).

### HOST PHYLOGENY

The method used to infer the host phylogeny has been described in detail elsewhere (Longdon et al. 2015). Briefly, publicly available sequences of the *28S*, *Adh*, *Amyrel*, *COI*, *COII*, *RpL32*, and *SOD* genes were collected from Genbank (see https://doi.org/10.6084/m9.figshare.13079366.v1 for a full breakdown of genes and accessions by species). Gene sequences were aligned in Geneious version 9.1.8 (https://www.geneious.com) using a progressive pairwise global alignment algorithm with free end gaps and a 70% similarity IUB cost matrix. Gap open penalties, gap extension penalties, and refinement iterations were kept as default.

Phylogenetic reconstruction was performed using BEAST version 1.10.4 (Drummond et al. 2012) as the subsequent phylogenetic mixed model (see below) requires a tree with the same root‐tip distances for all taxa. Genes were partitioned into separate ribosomal (*28S*), mitochondrial (*COI*, *COII*), and nuclear (*Adh*, *Amyrel*, *RpL32*, *SOD*) groups. The mitochondrial and nuclear groups were further partitioned into groups for codon position 1+2 and codon position 3, with unlinked substitution rates and base frequencies across codon positions. Each group was fitted to separate relaxed uncorrelated lognormal molecular clock models using random starting trees and four‐category gamma‐distributed HKY substitution models. The BEAST analysis was run twice, with 1 billion Markov chain Monte Carlo (MCMC) generations sampled every 100,000 iterations, using a birth‐death process tree‐shape prior. Model trace files were evaluated for chain convergence, sampling, and autocorrelation using Tracer version 1.7.1 (Rambaut et al. 2018). A maximum clade credibility tree was inferred from the posterior sample with a 10% burn‐in. The reconstructed tree was visualized using ggtree version 2.0.4 (Yu 2020).

### VIRUS ISOLATES

Virus stocks were kindly provided by Julien Martinez (DCV isolates) (Martinez et al. 2019), and Valérie Dorey and Maria Carla Saleh (CrPV) (van Rij et al. 2006). DCV‐C, DCV‐EB, and DCV‐M were originally isolated from fly stocks with origins in three separate continents; DCV‐C and DCV‐EB were isolated from lab stocks established by wild capture in Charolles, France and Ellis Beach, Australia, respectively, whereas DCV‐M was isolated directly from wild flies in Marrakesh, Morocco (Johnson and Christian 1999). The CrPV isolate was collected from *Teleogryllus commodus* in Victoria, Australia (Johnson and Christian 1996). Virus stocks were diluted in Ringers solution (Cold Spring Harbor Laboratory 2007) to equalize the relative concentrations of viral RNA and checked for contamination with CrPV (DCV isolates) and DCV (CrPV isolate) by qRT‐PCR as described below.

### VIRUS PHYLOGENY

Full genome sequences for DCV‐C (*MK645242*), DCV‐EB (*MK645239*), DCV‐M (*MK645243*), and CrPV (*NC_003924*) were retrieved from the NCBI Nucleotide database. Annotations of open reading frames (ORFs) for the replicase polyprotein (CrPV: *Q9IJX4*, DCV: *O36966*) and structural polyprotein (CrPV: *P13418*, DCV: *O36967*) were collected from the UniProtKB database and used to separate the coding and noncoding regions of each virus. ORF sequences were concatenated and aligned using the Geneious progressive pairwise translation alignment algorithm with a Blosum50 cost matrix and default parameters. Alignments were manually checked for quality and sequences aligning to CrPV ORF1 nucleotides 1–387 and 2704–2728 were removed due to the presence of large indels.

Phylogenetic reconstruction was performed using BEAST version 1.10.4 with translated ORF sequences fitted to an uncorrelated relaxed lognormal molecular clock model using a speciation birth‐death process tree‐shape prior. A Blosum62 substitution model (Henikoff and Henikoff 1992) with a gamma distribution of rate variation with four categories and a proportion of invariable sites was used. The model was run for 10 million MCMC generations sampled every 1000 iterations and evaluated in Tracer version 1.7.1 as above, and a maximum clade credibility tree inferred with a 10% burn‐in.

### INOCULATION

Before inoculation, 0‐ to 1‐day‐old male flies were kept in vials containing cornmeal media (Supporting Information Methods) and were transferred to fresh media every 2 days for 1 week. Male flies were chosen to avoid any effect of sex or of female mating status that has been shown to influence the susceptibility of females to infection with other pathogen types (Short and Lazzaro 2010; Duneau et al. 2017; Schwenke and Lazzaro 2017). Vials contained between 5 and 20 flies (mean = 14.5) and were kept at 22°C at 70% relative humidity in a 12‐hour light‐dark cycle. Flies were inoculated at 7–8 days old under CO_2_ anesthesia via septic pin prick with 12.5‐μm diameter stainless steel needles (Fine Science Tools, CA, USA). These needles were bent approximately 250 μm from the end to provide a depth stop and dipped in virus solution before being pricked into the pleural suture of each fly. Inoculation by this method has been shown to follow the same course as oral infection but is less stochastic (Landum et al., 2021). Inoculated flies were then snap frozen immediately in liquid nitrogen, providing a 0 days postinfection (dpi) time point, or maintained in cornmeal vials for a further 2 days ± 3 hours before freezing, providing a 2 dpi time point. Within replicate blocks, the 0 and 2 dpi vials for each virus were inoculated on the same day, and together constituted one biological replicate. We aimed to collect three biological replicates for each species and virus combination, with the order of species, vial (0 or 2 dpi), and virus randomized for each replicate block.

### MEASURING CHANGE IN VIRAL LOAD

To measure the change in viral load between 0 and 2 dpi, total RNA was extracted from flies homogenized in Trizol (Invitrogen, supplied by ThermoFisher) using chloroform‐isopropanol extraction, and reverse transcribed using Promega GoScript reverse transcriptase (Sigma) with random hexamer primers. qRT‐PCR was carried out on 1:10 diluted cDNA on an Applied Biosystems StepOnePlus system using Sensifast Hi‐Rox Sybr kit (Bioline). Cycle conditions were as follows: initial denaturation at 95°C for 120 seconds, then 40 cycles of 95°C for 5 seconds, and 60°C for 30 seconds.

DCV isolates were measured using the same primer pair (forward: 5′‐GACACTGCCTTTGATTAG‐3′; reverse: 5′‐CCCTCTGGGAACTAAATG‐3′) that targeted a conserved location and had similarly high efficiencies across all isolates. For CrPV, the following primers were used: forward, 5′‐TTGGCGTGGTAGTATGCGTAT‐3′; reverse, 5′‐TGTTCCGTCCTGCGTCTC‐3′. *RpL32* housekeeping gene primers varied by species (Tables SB and SC). For each sample, two technical replicates were performed for each amplicon (viral and *RpL32*).

Between‐plate variation in *C_t_* values was estimated and corrected for using a linear model with plate ID and biological replicate ID as parameters, as described elsewhere (Ruijter et al. 2006; Ruijter et al. 2015). Mean viral *C_t_* values from technical replicate pairs were normalized to *RpL32* and converted to fold‐change in viral load using the 2^–ΔΔ^ *^Ct^* method, where Δ*C_t_* = *C_t_*_:Virus_ – *C_t_*_:Rpl32_, and ΔΔ*C_t_* = Δ*C_t_*_:day0_ – Δ *C_t_*_:day2_.

Amplification of the correct products was verified by melt curve analysis. Repeated failure to amplify product, the presence of melt curve contaminants, or departures from the melt curve peaks of positive samples (±1.5°C for viral amplicons, ±3°C for *Rpl32*) in either the 0 or 2 dpi samples were used as exclusion criteria for biological replicates. In total, of the 180 unique combinations of host species and virus measured, three biological replicates were obtained for 161 combinations, two replicates for 18 combinations, and one replicate for one combination (*Drosophila virilis*, CrPV). Power analysis based on the downsampling of previous data has shown that this provides adequate statistical power to detect interactions between different experimental treatments and host species (Roberts and Longdon 2021).

### STATISTICAL ANALYSIS

Phylogenetic generalized linear mixed models were used to investigate the effects of host relatedness on viral load, and to examine correlations between the different virus isolates. Multivariate models were fitted using the R package MCMCglmm (Hadfield 2010) with the viral load of each virus isolate as the response variable. The structures of the models were as follows:

(1) $$y_{hiv}=\;\beta_{1:v}+\;\mu_{p:hv}+\mu_{s:hv}+e_{hiv},$$

(2) $$y_{hiv}=\;\beta_{1:v}+\;\mu_{p:hv}+e_{hiv}.$$

In these models, *y_hiv_* is the change in viral load for virus *v* in the *i^th^* biological replicate of host species *h*. The fixed effect *β*_1_ represents the intercepts for each virus isolate, the random effect *μ*_p_ represents the effects of the host phylogeny assuming a Brownian motion model of evolution, and *e* represents the model residuals. Model (1) also includes a species‐specific random effect that is independent of the host phylogeny (*μ*_s:_ *_hv_*). This explicitly estimates the nonphylogenetic component of between‐species variance and allows the proportion of variance explained by the host phylogeny to be calculated. *μ*_s:_ *_hv_* was removed from model (2) as model (1) struggled to separate the phylogenetic and species‐specific traits. Wing size, measured as the length of the IV longitudinal vein from the tip of the proximal segment to the join of the distal segment with vein V (Gilchrist et al. 2001), provided a proxy for body size (Huey et al. 2006) and was included in a further model as a fixed effect (*wingsizeβ_2:hv_*). This was done to ensure that any phylogenetic signal in body size did not explain the differences seen in viral load between species (Freckleton et al. 2002).

Within each of these models, the random effects and residuals were assumed to follow a multivariate normal distribution with a centered mean of 0 and a covariance structure of *V_p_ **⊗**A* for the phylogenetic effects, *V_s_ **⊗**I* for species‐specific effects, and *V_e_* **⊗** *I* for residuals, where **⊗** represents the Kronecker product. *A* represents the host phylogenetic relatedness matrix, *I* an identity matrix, and *V* represents 4 × 4 covariance matrices describing the between‐species variances and covariances of changes in viral load for the different viruses. Specifically, the matrices *V_p_* and *V_s_* describe the phylogenetic and nonphylogenetic between‐species variances in viral load for each virus and the covariances between them, whereas the residual covariance matrix *V_e_* describes within‐species variance that includes both true within‐species effects and measurement errors. Because each biological replicate was tested with a single virus isolate, the covariances of *V_e_* cannot be estimated and were set to 0.

Models were run for 13 million MCMC generations, sampled every 5000 iterations with a burn‐in of 3 million generations. Parameter expanded priors were placed on the covariance matrices, resulting in multivariate F distributions with marginal variance distributions scaled by 1000. Inverse‐gamma priors were placed on the residual variances, with a shape and scale equal to 0.002. To ensure the model outputs were robust to changes in prior distribution, models were also fitted with flat and inverse‐Wishart priors, which gave qualitatively similar results.

The proportion of the between species variance that can be explained by the phylogeny was calculated from model (1) using the equation *v_p_ /(v_p_ + v_s_)*, where *v_p_* and *v_s_* represent the phylogenetic and species‐specific components of between‐species variance (Freckleton et al. 2002), respectively, and are equivalent to phylogenetic heritability or Pagel's lambda (Pagel, 1999; Housworth et al. 2004). The repeatability of viral load measurements was calculated from model (2) as *v_p_/(v_p_ + v_e_)*, where *v_e_* is the residual variance of the model (Falconer 1996). Interspecific correlations in viral load were calculated from model (2) *v_p_* matrix as *cov_x_,_y_/√(var_x_ + var_y_)*. If correlations between viruses are close to 1 (with no change in the variance while the means remain constant), it would suggest there are no host species‐by‐virus interactions (Hudson et al. 2016). Parameter estimates reported are means of the posterior density, and 95% credible intervals (CIs) were taken to be the 95% highest posterior density intervals.

The data files and R scripts used in this study are available in an online repository: https://doi.org/10.6084/m9.figshare.13750711.v1.

## Results

### CHANGE IN VIRAL LOAD IS A REPEATABLE TRAIT AMONG HOST SPECIES

**Figure 1:**
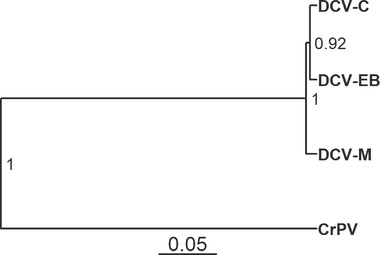
**Phylogeny of virus isolates**. Evolutionary relationships estimated from open reading frame (ORF) amino acid sequences presented in a midpoint‐rooted tree. Node labels represent the posterior probabilities of each clade, and the scale bar represents amino acid substitutions per site.

**Figure 2:**
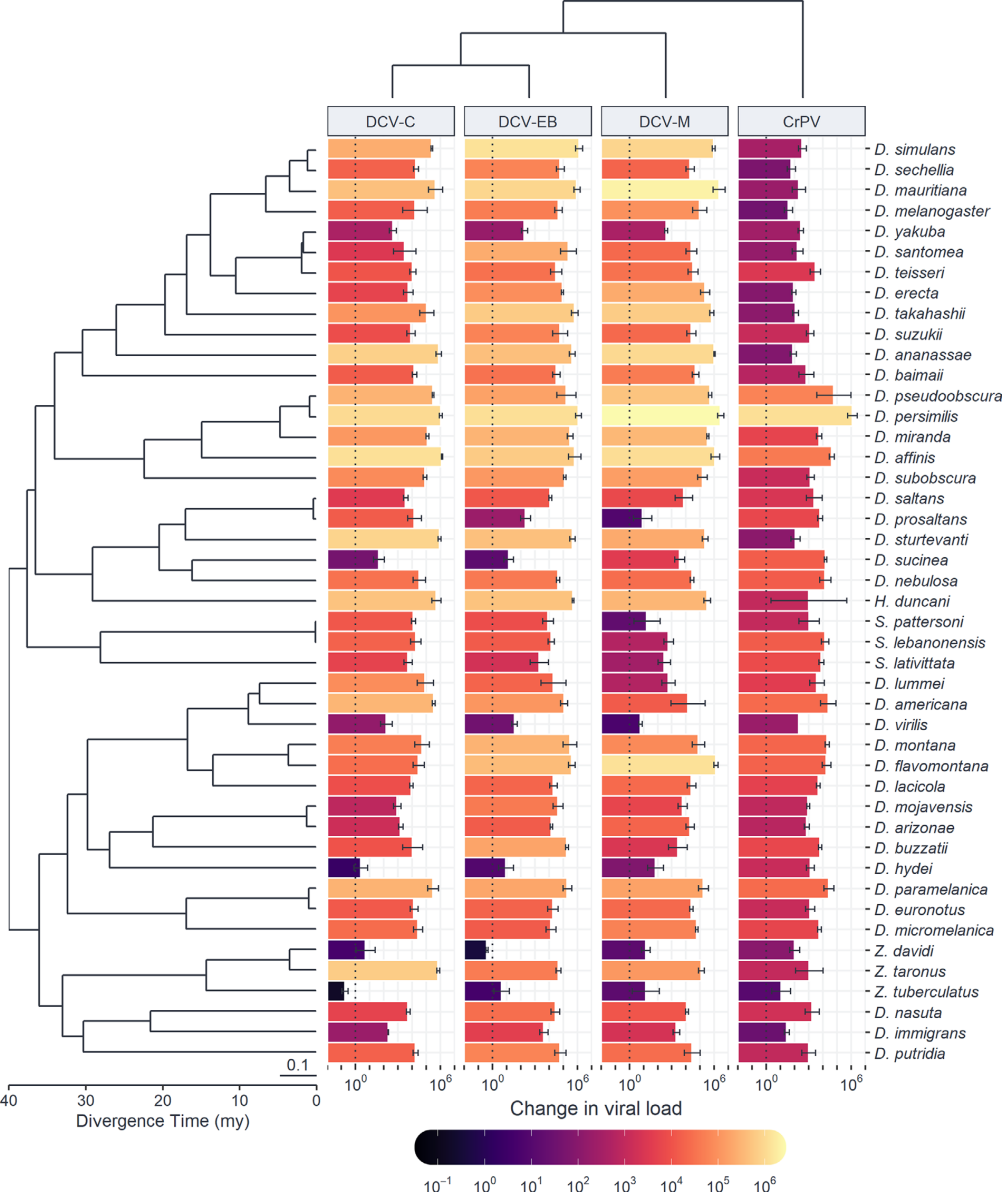
**Change in viral load across a diverse panel of *Drosophilidae* host species for different virus isolates**. Bar height and color show the mean change in viral load by 2 dpi on a log_10_ scale, with error bars representing the standard error of the mean. The phylogeny of *Drosophilidae* hosts is presented on the left, with the scale bar representing the number of nucleotide substitutions per site and scale axis representing the approximate age since divergence in millions of years (my) based on estimates from (Russo et al. 1995) and (Obbard et al. 2012). The virus cladogram, presented at the top, is based on the evolutionary relationships shown in Figure 1.

To investigate similarities between related viruses in the outcome of infection across host species, as well as the potential for different viruses to interact with host species effects, we experimentally infected 45 species of *Drosophilidae* with four virus isolates: DCV‐C, DCV‐EB, DCV‐M, and CrPV. The DCV isolates formed a distinct clade (>93% genome and ORF amino acid identity, with 265–556 SNPs between isolates), with the closest relationship between DCV‐C and DCV‐EB. CrPV formed an outgroup to the DCV isolates (57–59% identity, with over 4000 SNPs between CrPV and each DCV isolate; Fig. 1; Table 1). In total, 15,657 flies were inoculated, and the change in viral load after 2 days of infection was determined by qRT‐PCR (Fig. 2). The mean viral load within host species ranged from an approximately 2.7‐billion‐fold increase in *Drosophila persimilis* infected with DCV‐M to a 2.5‐fold decrease in *Zaprionus tuberculatus* infected with DCV‐C. Viral loads across host species tended to be higher for the DCV isolates, with a mean fold‐increase of roughly 11,000–19,000, and lower for CrPV, with a mean fold‐increase of roughly 1600.

**Table 1:** **Virus isolate sequence similarity**. Percentage sequence identity was calculated from multiple‐ alignment of whole genome nucleotides (white) or concatenated amino acid sequences of ORFs 1 and 2 (gray). Approximately 92 SNPs and 28 amino acid substitutions exist for every 1% of sequence divergence

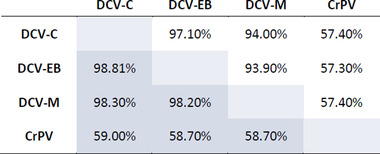

Phylogenetic generalized linear mixed models were fitted to the data to determine the proportion of variation in viral load explained by the host phylogeny (Table 2). The phylogeny explained 79% of the variation in viral load for CrPV but only 9–21% of the variation for the DCV isolates, with wide credible intervals on all the DCV estimates. This was due to the model struggling to separate phylogenetic and species‐specific effects for these viruses. The repeatability of viral load across host species was high for both CrPV (0.66) and the DCV isolates (0.92–0.96), with the between‐species phylogenetic component (*v_p_*) explaining a high proportion of the variation in viral load with little within‐species variation or measurement error (*v_r_*). We found no significant effect of wing length (a proxy for host body size) on viral load for any of the included viruses, with all estimates having credible intervals overlapping 0 (Table SD).

**Table 2:** **Estimates of mean change in viral load, repeatability, and the proportion of variation explained by the host phylogeny**. Estimates of the mean change in viral load and repeatability are taken from model (2), whereas estimates of the variation explained by the host phylogeny are taken from model (1)

Virus	Mean change in viral load	Repeatability	Variance explained by phylogeny
**DCV‐C**	11,585 (95% CI: 2304, 60,725)	0.96 (95% CI: 0.93, 0.98)	0.11 (95% CI: 0, 0.35)
**DCV‐EB**	19,083 (95% CI: 2740, 110,985)	0.96 (95% CI: 0.93, 0.98)	0.09 (95% CI: 0, 0.32)
**DCV‐M**	12,678 (95% CI: 1468, 98,648)	0.92 (95% CI: 0.87, 0.96)	0.23 (95% CI: 0, 0.51)
**CrPV**	1618 (95% CI: 385, 6472)	0.66 (95% CI: 0.46, 0.83)	0.79 (95% CI: 0.50, 1.00)

### CORRELATIONS BETWEEN VIRUSES ARE CONSISTENT WITH VIRUS BY HOST SPECIES INTERACTIONS

**Figure 3:**
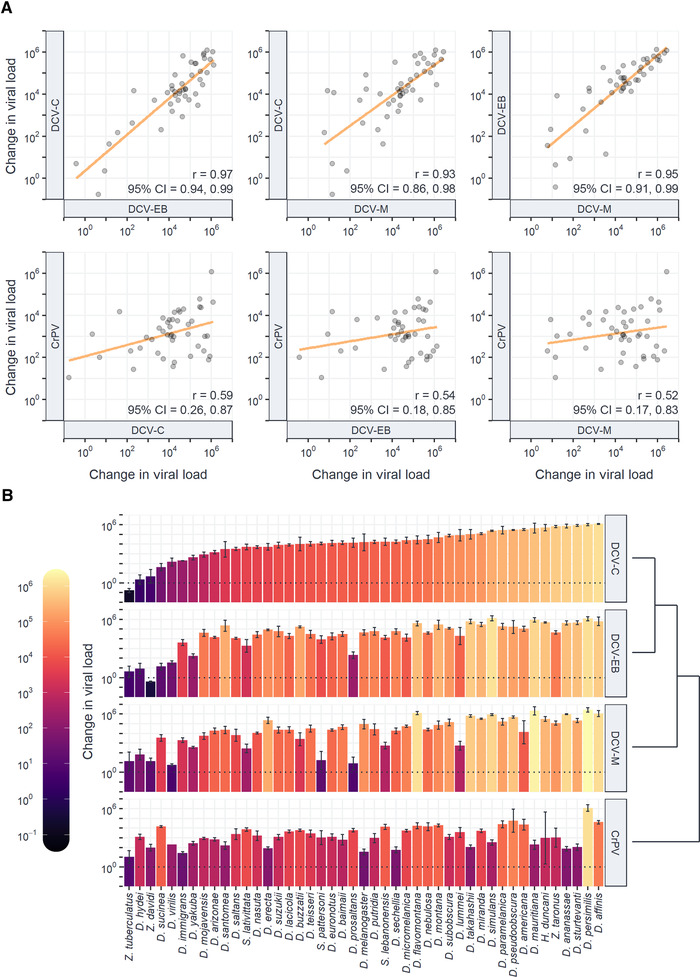
**Similarities in infection outcome across host species and interactions between virus and host species**. (A) Correlations in viral load between virus isolates. Individual points represent the mean change in viral load by 2 dpi for each host species on a log_10_ scale, and trend lines have been added from a univariate least‐squares linear model for illustrative purposes. Correlations (*r*) are the total interspecific correlations and 95% CIs from the output of model (2). (B) Differences in the rank order of host species susceptibility between virus isolates. Bar height and color show the mean change in viral load by 2 dpi on a log_10_ scale, with error bars representing the standard error of the mean. The order of species along the *x*‐axis has been sorted in ascending order of viral load during infection with DCV‐C. Deviations from this rank order of host species susceptibility for other viruses are indicative of crossing reaction norms and interactions between virus and host species. The virus cladogram is based on the evolutionary relationships shown in Figure 1.

Interspecific correlations in viral load between viruses were then estimated from the variance‐covariance matrices of model (2) (Fig. 3A). We found strong positive correlations between the DCV isolates (*r* > 0.93), with the strongest correlation between DCV‐C and DCV‐EB (*r* = 0.97). Correlations between DCV isolates and the more distantly related CrPV were positive (*r* = 0.52–0.59) but weaker than the correlations between the DCV isolates. The fact the DCV:CrPV correlations (and their 95% CIs) are not close to 1 is consistent with virus‐by‐host species interactions on viral load (Hudson et al. 2016). This is further demonstrated by the notable differences in the rank order of host species susceptibility for each virus (Fig. 3B), equivalent to a crossing over of reaction norms for the susceptibility of host species between different viruses (Ingleby et al. 2010).

DCV‐C appears to be slightly more strongly correlated to DCV‐EB than to DCV‐M (Δ*r* = 0.04; 95% CI: >0.001, 0.09; *P*_MCMC_ = 0.04), and more strongly correlated to DCV‐M than to CrPV (Δ*r* = 0.40; 95% CI: 0.18, 0.82, *P*_MCMC_ < 0.001), consistent with an increase in the strength of correlation between viruses with closer evolutionary relatedness. Point estimates imply a similar pattern for DCV‐EB, but the evidence for a stronger correlation with DCV‐C than DCV‐M was not well supported (Table SE).

## Discussion

Closely related host species present similar environments to novel viruses (Freckleton et al. 2002; Poulin et al. 2011), and so tend to share similar levels of susceptibility to a given virus (Gilbert and Webb 2007; Streicker et al. 2010; Longdon et al. 2011; Longdon et al. 2015; Farrell and Davies 2019; Guth et al. 2019; Mollentze et al. 2020). Likewise, closely related viruses are often assumed to share characteristics that make their host interactions, transmission, and evolutionary trajectories comparable (Chao et al. 2010; Hsieh 2010; Cann et al. 2013; Gomes et al. 2014; Du et al. 2017; Ruiz et al. 2017; Petersen et al. 2020; Zhu et al. 2020). Here, we measured the strength of correlations in viral load between four *Cripavirus* isolates across 45 host species of *Drosophilidae*, to look for similarities between related viruses as well as evidence of virus‐by‐host species interactions on the outcome of infection. We found positive correlations between every pair of viruses tested, indicating broad similarities in the outcome of infection across host species, but also evidence for interactions between virus and host species with changes in the rank order of host species susceptibility between the different viruses (Fig. 3). This highlights the importance of considering both host and virus traits in understanding the outcomes of virus host shifts.

The strong positive correlations between DCV isolates are likely due to relatively high levels of sequence conservation resulting in only small differences in their ability to infect different host species. However, in other viruses a small number of mutations have been shown to allow successful infections in novel hosts (Allison et al. 2016; de Vries et al. 2017). We find a few instances of such effects here. For example, in *Zaprionus davidi*, DCV‐EB shows a decline in viral load, suggesting it is failing to replicate and persist in this host species, whereas the other isolates show an increase in viral load in the same host. Similarly, *Scaptodrosophila pattersoni* is among the least susceptible to DCV‐M but has relatively high viral loads for the other virus isolates.

A greater number of these effects can be seen when comparing hosts infected with DCV isolates to those infected with CrPV, where multiple species have markedly different susceptibilities depending on the virus infecting them. For example, both *Drosophila ananassae* and *Drosophila sturtevanti* are within the five most susceptible species to DCV‐C, but also the eight least susceptible to CrPV. The weaker correlations that exist between DCV and CrPV may be due to interactions with different host traits that vary in their patterns across the host phylogeny. CrPV and DCV are known to have distinct methods of suppression of the host antiviral RNAi pathway (van Rij et al. 2006; Nayak et al. 2010) and cause pathology in different tissues (Cogni et al. 2016). Additionally, their relatively high levels of sequence divergence (57–59% identity) may have resulted in changes in the ability of each virus to bind to host cell receptors, use host replication machinery, or avoid host immune defences (Rothenburg and Brennan 2019).

The existence of correlations between viruses suggests that host susceptibility is not specific to individual viruses and that certain host clades may be broadly susceptible to infection. These hosts may share cell surface receptors with high affinity for both DCV and CrPV surface proteins, have a low efficiency or easily suppressed antiviral RNAi response, or have functionally diverged forms of other cellular processes linked to viral replication and persistence. Divergences in these immunological traits are possible candidates driving the large amount of variation in susceptibility we have detected across *Drosophilidae* host species. Host species that are permissive to multiple viruses and virus genotypes may allow for the persistence of increased genetic diversity in the virus population, allowing viruses to generate and maintain mutations that make them more likely to emerge in novel host species (Woolhouse et al. 2012; Woolhouse et al. 2014). They also have the potential to act as “mixing vessels,” providing increased opportunities for virus reassortment and recombination (Zhang et al. 2020), which has been proposed as a possible route for several viruses to acquire pandemic potential. (de Silva et al. 2012; Goldstein et al. 2021). Broadly susceptible host clades may therefore act as common reservoirs and donors of emerging infectious diseases and identifying them in relevant systems could inform control and prevention strategies (Streicker and Gilbert 2020).

The differences in correlation strength between pairs of viruses tended to follow differences in their evolutionary divergence, such that more closely related pairs of viruses were more strongly correlated in the outcome of infection across host species. This provides some tentative evidence that the ability of a virus to infect a novel host may be inferred based on its evolutionary relatedness to other viruses. A greater number of more diverged isolates from this virus family would have allowed this potential phylogenetic effect to be investigated more conclusively, although to our knowledge the viruses included here represent the most diverged viruses of this genus that are readily available for study. The pathogen phylogenetic effects seen here have also been observed in other pathogen and parasite systems (Vienne et al. 2009; Hadfield et al. 2014), including genetic distance effects seen in other *Drosophila* parasites (Perlman and Jaenike 2003). However, the rapid mutation rates and small genomes of RNA viruses may cause these effects to exist, and become perturbed, across shorter time scales than for other pathogens. Numerous examples exist where a small number of genetic changes in viruses cause large phenotypic differences (Tsetsarkin et al. 2007; Allison et al., 2016; de Vries et al. 2017; Wong et al., 2018), which would be exceptions to any link between correlation strength and evolutionary relatedness (Housworth et al. 2004).

Nevertheless, virus phylogenetic effects may still prove to be a useful proxy for determining the likelihood of novel virus emergence. Further work is now needed to expand the findings of this study to broader groups of viruses, and to test the importance of the virus phylogeny in determining the potential outcomes of virus host shifts.

## Supporting information

Supplement Material
