# Supplementary material for: Between virus correlations in the outcome of infection across host species: Evidence of virus by host species interactions
Source: Evol Lett. 2021 Jul 15;5(5):472–83. doi: 10.1002/evl3.247 (PMC8484721; doi:10.1002/evl3.247)
Supplement: Supplementary file 1 — Supplement Material [file EVL3-5-472-s001.pdf]

S1 Text

S1 Text Supplementary Methods.

Drosophila Media Recipes.

To prepare media, ingredients (excluding nipagin and proprionic acid) are mixed together and brought to boil, then allowed to cool to 80°C before adding preservatives and pouring into bottles. Media is allowed to solidify overnight at room temperature before use.

Banana:

Ingredient	Quantity
**Water**	1,000ml
**Agar**	10g
**Yeast**	30g
**Bananas***	150g
**Malt Extract**	30g
**Molasses**	50g
**Nipagin**	25ml

* Blended in water before boiling.

Cornmeal:

Ingredient	Quantity
**Water**	1,000ml
**Agar**	11g
**Yeast**	19g
**Cornmeal**	88g
**Dextrose**	88g
**Nipagin**	29ml

Malt:

Ingredient	Quantity
**Water**	1,000ml
**Agar**	10g
**Yeast**	20g
**Semolina**	60g
**Malt Extract**	80g
**Nipagin**	14ml
**Propionic Acid**	5ml

Propionic:

Ingredient	Quantity
**Water**	1,000ml
**Agar**	10g
**Yeast**	20g
**Cornmeal**	70g
**Soya Flour**	10g
**Malt Extract**	80g
**Molasses**	22g
**Nipagin**	14ml
**Propionic Acid**	6.2ml

S1 Text Supplementary Tables

S1 Text Table A. Full list of Drosophila host species used in this study, including wingspan data used as a proxy for body size, the media type used to maintain each stock population, and whether the stocks originate from isofemale lines. Recipes for each media type can be found below.

Species	Genus	Wingsize (mm)	Media	Isofemale
D. affinis	*Drosophila*	1.803	Malt	TRUE
D. americana	*Drosophila*	2.045	Malt	TRUE
D. ananassae	*Drosophila*	1.493	Cornmeal	TRUE
D. arizonae	*Drosophila*	1.548	Banana	TRUE
D. baimaii	*Drosophila*	1.561	Cornmeal	TRUE
D. buzzatii	*Drosophila*	1.902	Malt	TRUE
D. erecta	*Drosophila*	1.581	Malt + yeast	TRUE
D. euronotus	*Drosophila*	2.222	Cornmeal	FALSE
D. flavomontana	*Drosophila*	2.192	Malt + yeast	TRUE
D. hydei	*Drosophila*	2.182	Cornmeal	TRUE
D. immigrans	*Drosophila*	2.153	Malt + yeast	TRUE
D. lacicola	*Drosophila*	2.268	Malt	TRUE
D. lummei	*Drosophila*	2.558	Malt + yeast	TRUE
D. mauritiana	*Drosophila*	1.507	Proprionic	TRUE
D. melanogaster	*Drosophila*	1.716	Cornmeal	TRUE
D. micromelanica	*Drosophila*	1.895	Cornmeal	TRUE
D. miranda	*Drosophila*	2.395	Cornmeal	TRUE
D. mojavensis	*Drosophila*	1.650	Banana	TRUE
D. montana	*Drosophila*	2.706	Malt + yeast	TRUE
D. nasuta	*Drosophila*	1.917	Cornmeal	TRUE
D. nebulosa	*Drosophila*	1.826	Cornmeal	TRUE
D. paramelanica	*Drosophila*	1.946	Cornmeal	TRUE
D. persimilis	*Drosophila*	2.013	Malt	TRUE
D. prosaltans	*Drosophila*	1.699	Proprionic	TRUE
D. pseudoobscura	*Drosophila*	1.863	Malt	TRUE
D. putridia	*Drosophila*	1.639	Proprionic	TRUE
D. saltans	*Drosophila*	1.600	Proprionic	TRUE
D. santomea	*Drosophila*	1.489	Cornmeal	TRUE
D. sechellia	*Drosophila*	1.424	Proprionic	TRUE
D. simulans	*Drosophila*	1.484	Cornmeal	TRUE
D. sturtevanti	*Drosophila*	1.779	Cornmeal	TRUE
D. subobscura	*Drosophila*	2.056	Cornmeal	TRUE
D. sucinea	*Drosophila*	1.932	Cornmeal	TRUE
D. suzukii	*Drosophila*	2.100	Cornmeal	TRUE
D. takahashii	*Drosophila*	1.559	Cornmeal	TRUE
D. teisseri	*Drosophila*	1.463	Cornmeal	TRUE
D. virilis	*Drosophila*	2.253	Proprionic	TRUE
D. yakuba	*Drosophila*	1.307	Cornmeal	TRUE
H. duncani	*Hirtodrosophila*	1.969	Proprionic	TRUE
S. lativittata	*Scaptodrosophila*	1.851	Banana	TRUE
S. lebanonensis	*Scaptodrosophila*	2.053	Proprionic	TRUE
S. pattersoni	*Scaptodrosophila*	2.023	Banana	TRUE
Z. davidi	*Zaprionous*	1.911	Banana	TRUE
Z. taronus	*Zaprionous*	2.131	Banana	TRUE
Z. tuberculatus	*Zaprionous*	1.914	Banana	TRUE

Cornmeal and proprionic media are dusted with yeast before use. Malt and banana media are left bare unless otherwise stated.

S1 Text Table B. *RPL32* primer sequences used to amplify the host housekeeping gene. Primers were designed to amplify across an intron boundary to ensure only cDNA sequences were amplified. Different primer combinations were used for different host species (S1 Text Table C) to account for SNPs in the primer binding sites.

	Name	Sequence
**Forward**	RpL32_qPCR_F-a	TGCCAAGTTGTCGCACAAATGG
	RpL32_qPCR_F-b	TGCTAAGTTGTCGCACAAATGG
	RpL32_qPCR_F-c	TGCCAAGCTGTCGCACAAATGG
	RpL32_qPCR_F-d	TGCTAAGCTGTCGCACAAATGG
	RpL32_qPCR_F-e	TGCGAAGTTGTCGCACAAATGG
	RpL32_qPCR_F-f	TGCGAAGCTGTCGCACAAATGG
**Reverse**	RpL32_qPCR_R-a	TGCGCTTGTTGGAACCGTAAC
	RpL32_qPCR_R-b	TGCGCTTGTTGGATCCGTAAC
	RpL32_qPCR_R-c	TGCGCTTGTTGGAACCATAAC
	RpL32_qPCR_R-d	TGCGCTTGTTGGAGCCGTAAC
	RpL32_qPCR_R-e	TGCGCTTGTTAGAACCGTAAC
	RpL32_qPCR_R-f	TACGCTTGTTGGAACCGTAAC
	RpL32_qPCR_R-g	TGCGCTTGTTGGAACCGTAGC
	RpL32_qPCR_R-h	TGCGCTTGTTCGATCCGTAAC
	RpL32_qPCR_R-i	TGCGCTTGTTGGAGCCATAAC
	RpL32_qPCR_R-j	TGCGCTTGTTTGATCCGTAAC
	RpL32_qPCR_R-k	TGCGCTTGTTTGAACCATAAC
	RpL32_qPCR_R-l	TACGCTTGTTGGAACCATAAC
	RpL32_qPCR_R-m	TACGCTTGTTGGAGCCGTAAC
	RpL32_qPCR_R-n	TGCGCTGGTTGGAACCATAAC
	RpL32_qPCR_R-o	TGAGCTTGTTCGATCCGTAAC
	RpL32_qPCR_R-p	TACGCTTGTTGGAGCCATAAC
	RpL32_qPCR_R-q	TGAGCTTGTTTGATCCGTAAC
	RpL32_qPCR_R-r	TAAGCTTGTTGGATCCGTAGC
	RpL32_qPCR_R-s	TCAGCTTGTTGGATCCATAGC

S1 Text Table C. *RPL32* primer combinations used for each host species.

Species	Forward	Reverse
D. affinis	F-a	R-i
D. americana	F-c	R-a
D. ananassae	F-f	R-a
D. arizonae	F-a	R-a
D. baimaii	F-a	R-r
D. buzzati	F-a	R-e
D. erecta	F-d	R-h
D. euronotus	F-a	R-g
D. flavomontana	F-c	R-a
D. hydei	F-a	R-a
D. immigrans	F-b	R-p
D. lacicola	F-c	R-a
D. lummei	F-c	R-a
D. mauritiana	F-d	R-h
D. melanogaster	F-d	R-h
D. micromelanica	F-a	R-g
D. miranda	F-a	R-d
D. mojavensis	F-a	R-a
D. montana	F-c	R-a
D. nasuta	F-b	R-f
D. nebulosa	F-b	R-c
D. paramelanica	F-a	R-g
D. persimilis	F-a	R-b
D. prosaltans	F-a	R-n
D. pseudoobscura	F-a	R-m
D. putridia	F-d	R-q
D. saltans	F-a	R-n
D. santomea	F-a	R-n
D. sechellia	F-d	R-h
D. simulans	F-d	R-h
D. sturtevanti	F-a	R-l
D. subobscura	F-a	R-i
D. sucinea	F-b	R-k
D. suzukii	F-d	R-o
D. takahashii	F-d	R-o
D. teissieri	F-d	R-h
D. virilis	F-c	R-a
D. yakuba	F-d	R-h
H. duncani	F-f	R-c
S. lativittata	F-a	R-m
S. lebanonensis	F-d	R-h
S. pattersoni	F-a	R-m
Z. davidi	F-a	R-c
Z. taronus	F-a	R-c
Z. tuberculatus	F-a	R-c

S1 Text Table D. MCMCglmm model estimates of the effects of wing length on viral load of each virus across host species.

Virus	Posterior Mean	95% CI
CrPV	0.74	-10.05, 10.56
DCV-C	1.01	-8.59. 11.45
DCV-EB	0.34	-9.71, 10.16
DCV-M	1.04	-8.50, 11.22

S1 Text Table E. Estimates of the differences between correlations in viral load (Δr). The differences presented have all been calculated as column minus row. Evidence for significant differences between correlations are shown as the mean and 95% CIs for the posterior distribution of the differences in correlations (white) and as P_{MCMC} (grey). P_{MCMC} corresponds to 2 * P_{min}, where P_{min} is either the probability of iterations in the posterior density being positive or negative, whichever is smaller. Significant differences (95% CIs not crossing zero, P_{MCMC} < 0.05) are highlighted in bold.

	DCV-C : DCV-EB	DCV-C : DCV-M	DCV-EB : DCV-M	CrPV : DCV-C	CrPV : DCV-EB	CrPV : DCV-M
**DCV-C : DCV-EB**		**-0.04 (95% CI = -0.09, 0.00)**	-0.02 (95% CI = -0.07, 0.02)	**-0.37 (95% CI = -0.70, -0.13)**	**-0.43 (95% CI = -0.78, -0.16)**	**-0.45 (95% CI = -0.82, -0.18)**
**DCV-C : DCV-M**	**0.04 (p = 0.03)**		0.03 (95% CI = -0.01, 0.08)	**-0.33 (95% CI = -0.68, -0.10)**	**-0.38 (95% CI = -0.77, -0.13)**	**-0.40 (95% CI = -0.77, -0.16)**
**DCV-EB : DCV-M**	0.02 (p = 0.47)	-0.03 (p = 0.17)		**-0.36 (95% CI = -0.69, -0.11)**	**-0.41 (95% CI = -0.73, -0.09)**	**-0.43 (95% CI = -0.78, -0.16)**
**CrPV : DCV-C**	**0.37 (p < 0.001)**	**0.33 (p < 0.001)**	**0.36 (p < 0.001)**		-0.05 (95% CI = -0.14, 0.03)	-0.07 (95% CI = -0.23, 0.06)
**CrPV : DCV-EB**	**0.43 (p < 0.001)**	**0.38 (p < 0.001)**	**0.41 (p < 0.001)**	0.05 (p = 0.24)		-0.02 (95% CI = -0.17, 0.11)
**CrPV : DCV-M**	**0.45 (p < 0.001)**	**0.40 (p < 0.001)**	**0.43 (p < 0.001)**	0.07 (p = 0.32)	0.02 (p = 0.76)	
